# Pharmacologic inhibition of HNF4α prevents parenteral nutrition associated cholestasis in mice

**DOI:** 10.1038/s41598-023-33994-3

**Published:** 2023-05-12

**Authors:** Swati Ghosh, Michael W. Devereaux, David J. Orlicky, Ronald J. Sokol

**Affiliations:** 1 Section of Pediatric Gastroenterology, Hepatology and Nutrition, Department of Pediatrics, Pediatric Liver Center, Digestive Health Institute, Children’s Hospital Colorado, University of Colorado School of Medicine, 13123 E. 16th Ave, Aurora, CO 80045 USA; 2 Department of Pathology, University of Colorado School of Medicine, 12801, E 17th Ave, Aurora, CO 80045 USA

**Keywords:** Cytokines, Immunology, Molecular biology, Diseases, Gastroenterology

## Abstract

Prolonged parenteral nutrition (PN) can lead to PN associated cholestasis (PNAC). Intestinally derived lipopolysaccharides and infused PN phytosterols lead to activation of NFκB, a key factor in PNAC. Our objective was to determine if inhibition of HNF4α could interfere with NFκB to alleviate murine PNAC. We showed that HNF4α antagonist BI6015 (20 mg/kg/day) in DSS-PN (oral DSS x4d followed by Total PN x14d) mice prevented the increased AST, ALT, bilirubin and bile acids and reversed mRNA suppression of hepatocyte *Abcg5/8, Abcb11,* FXR, SHP and MRP2 that were present during PNAC. Further, NFκB phosphorylation in hepatocytes and its binding to LRH-1 and BSEP promoters in liver, which are upregulated in DSS-PN mice, were inhibited by BI6015 treatment. BI6015 also prevented the upregulation in liver macrophages of *Adgre1 *(F4/80) and *Itgam* (CD11B) that occurs in DSS-PN mice, with concomitant induction of anti-inflammatory genes (*Klf2, Klf4, Clec7a1, Retnla*). In conclusion, HNF4α antagonism attenuates PNAC by suppressing NFκB activation and signaling while inducing hepatocyte FXR and LRH-1 and their downstream bile and sterol transporters. These data identify HNF4α antagonism as a potential therapeutic target for prevention and treatment of PNAC.

## Introduction

Advances made in the administration of parenteral nutrition (PN) have improved the clinical care of patients with short bowel syndrome and other causes of intestinal failure (IF) in children. Nevertheless, the complication of PN-associated cholestatic liver disease (PNAC; also called IF associated liver disease or IFALD) that may progress to liver failure has been one of the major challenges in managing patients on prolonged PN. PNAC is characterized by intrahepatic cholestasis and worsening of liver function in patients having parenteral nutrition for extended periods of time (signs may emerge as quick as within the first two weeks of administration of parenteral nutrition). The disorder commonly happens in neonates and usually resolves with transition to enteral feeding, although severe cases associated with IF may progress to liver fibrosis, cirrhosis, and portal hypertension^1–5^. The pathogenesis of PNAC is postulated to involve toxic components of the PN solution, bacterial overgrowth and dysbiosis of the small intestine, impaired intestinal barrier function, sepsis-associated cholestasis, altered bile acid composition, and other factors^1,6–9^.

Components of plant-based intravenous lipid emulsions have been associated with PNAC both in humans with IFALD^6,10^ and in animal models^10–12^. We have demonstrated that accumulation of soybean lipid emulsion-derived plant sterols and intestinally absorbed lipopolysaccharides (LPS) likely play major synergistic roles in the pathogenesis of PNAC based on a mouse model that combines intestinal injury leading to increased permeability and dysbiosis with total parenteral nutrition^13^.

Hepatocyte nuclear factor 4-α (HNF4α), encoded by *NR2A1*, is a member of the nuclear receptor superfamily of ligand-dependent transcription factors and is expressed in liver and gastrointestinal organs (pancreas, stomach, and intestine)^13^ where it plays a vital role in hepatic fatty acid metabolism and bile acid synthesis^13–15^. Dysregulation of HNF4α expression has been observed in many human diseases including non-alcoholic steatohepatitis, ulcerative colitis, colon cancer, maturity-onset diabetes of the young, liver cirrhosis, and hepatocellular carcinoma^13–15^, however, its precise role in the pathogenesis of these disorders is not well understood.

Recently, we demonstrated in a PNAC mouse model the essential role of macrophage derived-IL-1β in activating and inducing the phosphorylation of NFκB, leading to down regulation of hepatocyte bile and sterol transporters. *Il1r*^*−/−*^, as well as anakinra-treated, mice were protected from PNAC, implicating IL-1β in PNAC pathogenesis^10,16^. NFκB is a protein complex that regulates transcription of DNA, cytokine production and cell survival, therefore, pharmacologically targeting NFκB in PNAC would be challenging and may have off target detrimental effects. In silico analysis showed that NFκB and HNF4α share the same DNA binding site in promoters of several genes involved in bile acid pathways (*CYP27A1* and *PVMK*) genes. Moreover, HNF4α has been shown to play a role in NFκB signaling in a hepatoma cell-line^17^. Therefore, pharmacologic inhibition of HNF4α may be a preferred method for preventing NFκB activation in PNAC. Here we demonstrate upregulation of HNF4α in PNAC and that pharmacologic antagonism of HNF4α (by BI6015) significantly reduced hepatic NFκB activation, induced hepatocyte bile and sterol transporter expression, and reduced cholestasis and hepatic injury in murine PNAC.

## Results

### HNF4α upregulation in PNAC and reduced liver injury and cholestasis by HNF4α antagonism

**Figure 1:**
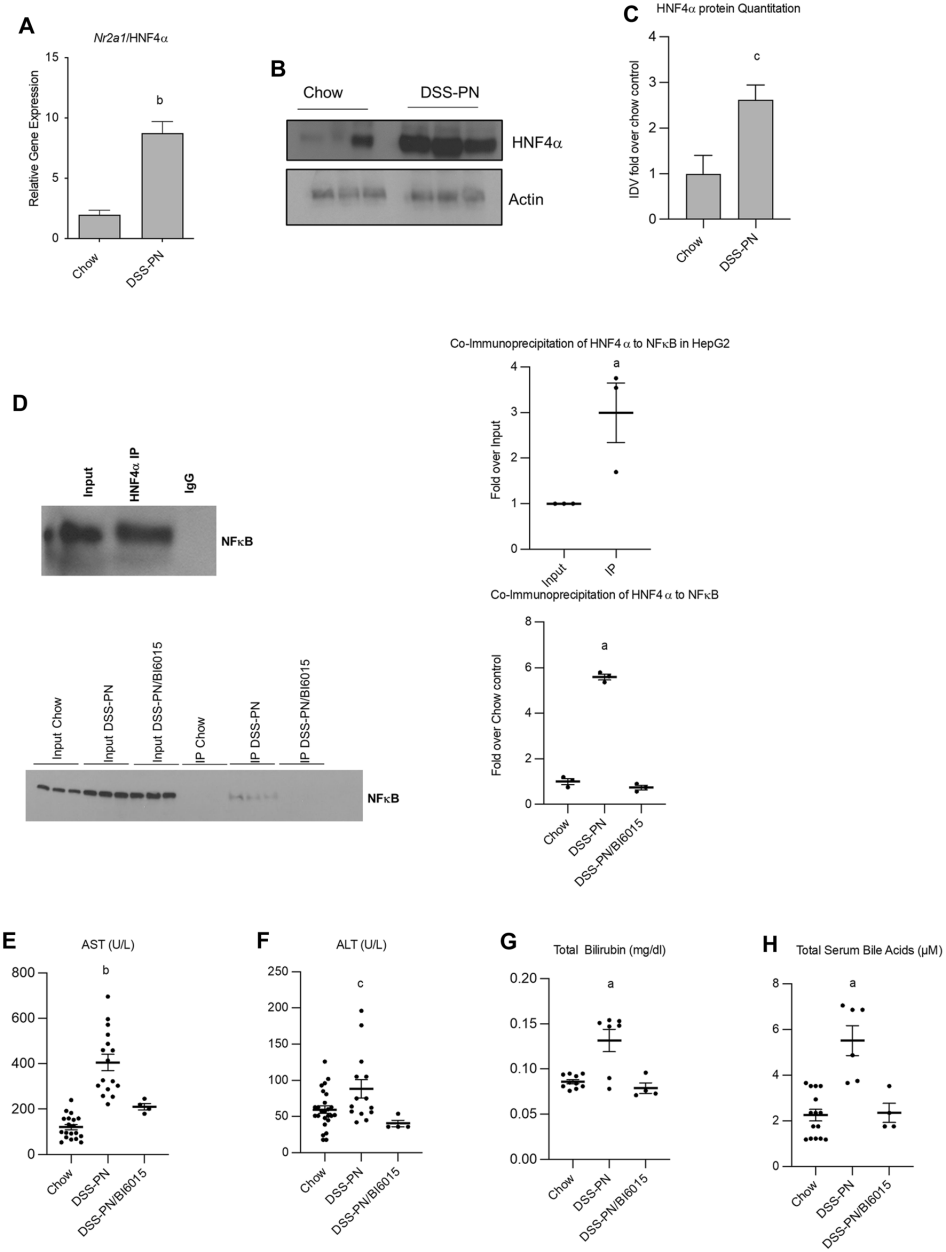
HNF4α expression and effect of its antagonism in PNAC mouse model. (**A**) Gene expression analysis of hepatic *Nr2a2/*HNF4α from Chow and DSS-PN (PNAC) treated mice. mRNA expression was determined after normalization to *Hprt1* as an endogenous control gene and expressed relative to results obtained from untreated Chow controls. Data are from 3 Chow and 3 DSS-PN mice. (**B**) Western analysis of HNF4α protein in liver homogenate from Chow and DSS-PN mice. Data are from 3 Chow and 3 DSS-PN mice (**C**) Quantification of integrated density values (IDV) normalized to actin and expressed relative to Chow control. (**D**) Upper panel: Co-immunoprecipitation of HNF4α showed that NFκB binds to HNF4α in HepG2 cells. Densitometry (IDV) of representative blot of 3 immunoblots. Lower panel: Co-immunoprecipitation of HNF4α in liver homogenate of experimental mice showed that NFκB binding to HNF4a was significantly higher in DSS-PN mice than chow or DSS-PN/BI6015 treated mice. Densitometry (IDV) of immunoblots is shown from 3 Chow, 3 DSS-PN and 3 DSS-PN/BI6015 mice. (**E**) Serum aspartate aminotransferase (AST). Data are from 19 Chow,16 DSS-PN and 4 DSS-PN/BI6015 (HNF4α antagonist) mice. (**F**) Serum alanine aminotransferase (ALT). Data are from 23 Chow,14 DSS-PN and 4 DSS-PN/BI6015 mice. (**G**) Total serum bilirubin. Data are from 10 Chow,7 DSS-PN and 4 DSS-PN/BI6015 mice. (**H**) Total serum bile acids. Data are from 15 Chow, 6 DSS-PN and 4 DSS-PN/BI6015 mice. Data expressed as mean + /− SEM, data points represent individual mice. Statistical analysis was performed, and adjusted P values obtained using (**B**, **C**, **D**, **E**–**H**) one-way ANOVA with Tukey’s correction for multiple comparisons and (**A**–**D**) by Student’s unpaired *t*-test. ^a^*p* < 0.0001; ^b^*p* < 0.01 or ^c^*p* < 0.05 versus all other groups.

First, we analyzed mRNA expression and protein expression of HNF4α (Fig. 1A–C) in liver from the PNAC mouse model (oral dextran sulfate sodium [DSS; MP Biomedicals, Santa Ana, CA] pretreated mice for 4 days followed by PN for 14 days [DSS-PN]) and show that HNF4α is upregulated in PNAC. We have previously reported the increased activation of NFκB in liver of DSS-PN mice^16^. In silico analysis showed that NFκB and HNF4α share a common binding site in promoters of several genes involved in bile acid pathways (*CYP27A1* and *PVMK*). To determine the effect of interaction, we performed coimmunoprecipitation in HepG2 cells and found that HNF4α interacts significantly with NFκB (Fig. 1D). To determine the translational significance of this interaction, we treated the PNAC mouse model with a pharmacologic antagonist of HNF4α, BI6015, 20 mg/kg/body weight per day, infused in the PN solution from day 4 through day 14 of PN. We then performed coimmunoprecipitation in liver homogenate from chow, DSS-PN and DSS-PN/BI6015 mice and found that HNF4α interacted significantly with NFκB in DSS-PN group compared to others (Fig. 1D). Previously, we reported that serum AST, ALT, total bilirubin and bile acid levels were all significantly increased in DSS-PN mice^16,18^. Compared to DSS-PN mice at day 14, DSS-PN/BI6015 mice had significantly reduced serum AST, ALT, bilirubin and total serum bile acids that were comparable to Chow mice controls (Fig. 1E–H). Thus, treatment with the HNF4α antagonist BI6015 reversed the evidence of hepatocyte injury and cholestasis during murine PNAC.

### HNF4α antagonism restores expression of FXR, FXR target genes, and sterol transporters during PNAC

**Figure 2:**
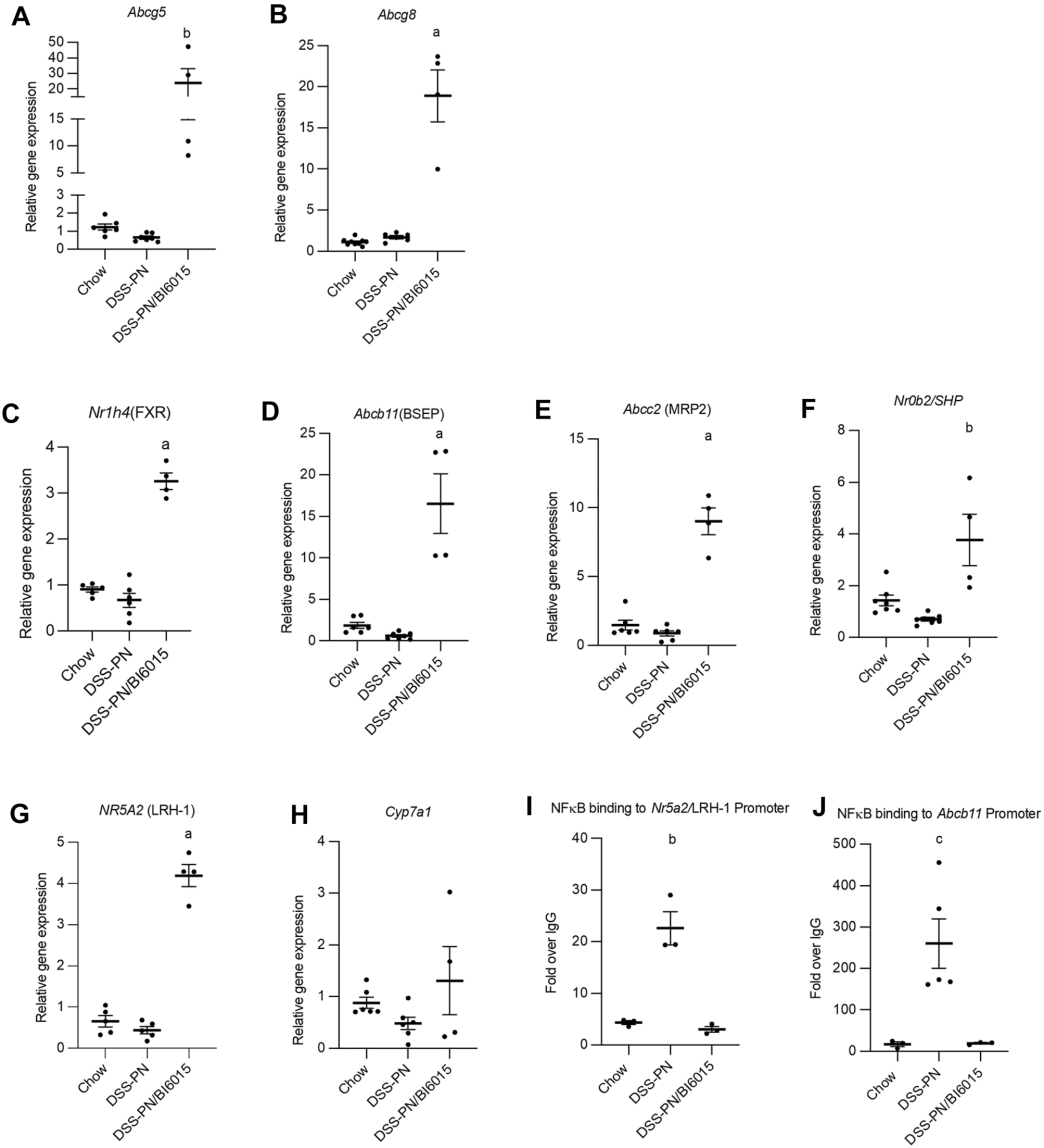
HNF4α antagonist BI6015 induces expression of sterol transporters, and FXR and its target genes in PNAC mouse model. qRT-PCR analysis of isolated hepatocytes from Chow, DSS-PN and DSS-PN/BI6015 mice for (**A**) *Abcg5*, data are from 5 Chow,7 DSS-PN and 4 DSS-PN/BI6015 mice (**B**) *Abcg8*, data are from 9 Chow, 6 DSS-PN and 4 DSS-PN/BI6015 mice (**C**) *Nr1h4* (FXR), data are from 5 Chow, 6 DSS-PN and 4 DSS-PN/BI6015 mice (**D**) *Abcb11* (BSEP), data are from 6 Chow, 7 DSS-PN and 4 DSS-PN/BI6015 mice (**E**) *Abcc2* (MRP2), data are from 6 Chow, 6 DSS-PN and 4 DSS-PN/BI6015 mice (**F**) *Nr0B2* (SHP), data are from 7 Chow, 7 DSS-PN and 4 DSS-PN/BI6015 mice (**G**) *Nr5a2* (LRH-1), data are from 5 Chow, 5 DSS-PN and 4 DSS-PN/BI6015 mice and (**H**) *Cyp7a1*, data are from 6 Chow, 6 DSS-PN and 4 DSS-PN/BI6015 mice. Chromatin immunoprecipitation (ChIP) of liver homogenate for NFκB binding to the promoter regions of (**I**) *Nr5a2*, data are from 3 Chow, 3DSS-PN and 3 DSS-PN/BI6015 mice and (**J**) *Abcb11*, data are from 3 Chow, 5 DSS-PN and 3 DSS-PN/BI6015 mice using an NFκB -p65 specific antibody. Data points represent individual mice and are expressed as mean + /− SEM. Statistical analysis performed by one-way ANOVA with Tukey’s correction for multiple comparisons. ^a^*p* < 0.0001; ^b^*p* < 0.01 or ^c^*p* < 0.05 versus all other groups.

We previously reported that the use of soy-oil (SO)-based lipid emulsions in the DSS-PN mouse was strongly associated with a relative reduction of hepatic mRNA expression of the canalicular sterol transporters, *Abcg5 and Abcg8*, as well as LRH-1 (*Nr5a2*) and FXR (*Nr1h4)* and its target genes coding for SHP (*Nrob2*), BSEP (*Abcb11*) and MRP2 (*Abcc2*), which preceded the onset of hepatic injury and cholestasis^16,18^. To determine the impact of HNF4α antagonism on expression of these genes and the gene regulating the rate-limiting step in bile acid synthesis, *Cyp7A1*, the relative mRNA expression was determined using qRT-PCR analysis of hepatocytes isolated from experimental mice. BI6015 treatment starting on day 4 of PN in DSS-PN mice (when hepatic macrophages have been shown to already be activated)^10^ significantly increased hepatocyte mRNA levels at day 14 of *Abcg5*, *Abcg8*, *Nr1h4, Abcb11*, *Abcc2, Nr0b2* and *Nr5a2* (which were reduced in DSS-PN mice) to amounts exceeding chow controls as well as DSS-PN mice (Fig. 2A–G). BI6015 treatment did not show any significant effects on *Cyp7a1* expression (Fig. 2H). Thus, pharmacological antagonism of HNF4α in vivo was able to significantly induce transcription of hepatocyte genes involved in phytosterol export and regulating canalicular bile secretion, even above the levels observed in the chow control mice.

We next examined the effect of HNF4α antagonism on binding of NFκB to its response element on the promoters of *Nr5a2*/LRH-1 and *Abcb11*/BSEP by chromatin immunoprecipitation (ChIP) assay in liver from experimental mice. BI6015 treatment in PNAC mice significantly decreased NFκB binding to the *Nr5a2* and *Abcb11* promoters in liver compared to DSS-PN mice (Fig. 2I–J). In our previous work we reported that the binding of NFκB to promoters of hepatocyte genes encoding canalicular transporters of bile acids, and sterols interfered with FXR and LRH-1 upregulation of these genes during PNAC, resulting in cholestasis^10,16^. Combined with the current study, these findings indicate that pharmacologic antagonism of HNF4α suppresses NFκB binding to promoters in genes critical for canalicular bile export, and at least in part results in increased expression of BSEP, thus reducing exposure of the hepatocyte to toxic levels of bile acids and protecting the DSS-PN mice from cholestasis and hepatic injury.

### HNF4α antagonism reduced proinflammatory gene activation and induced anti-inflammatory genes in intrahepatic mononuclear cells and BMDMs

**Figure 3:**
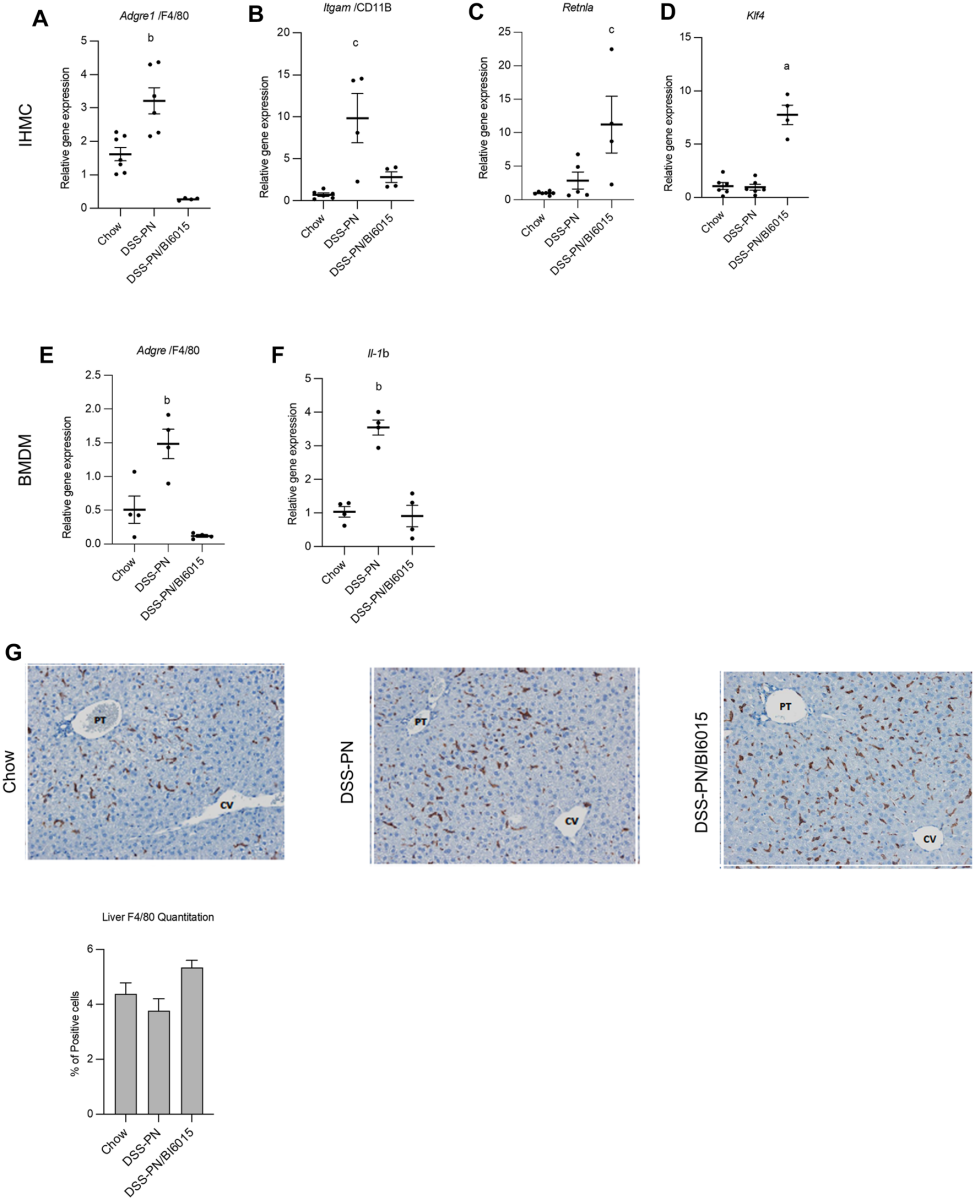

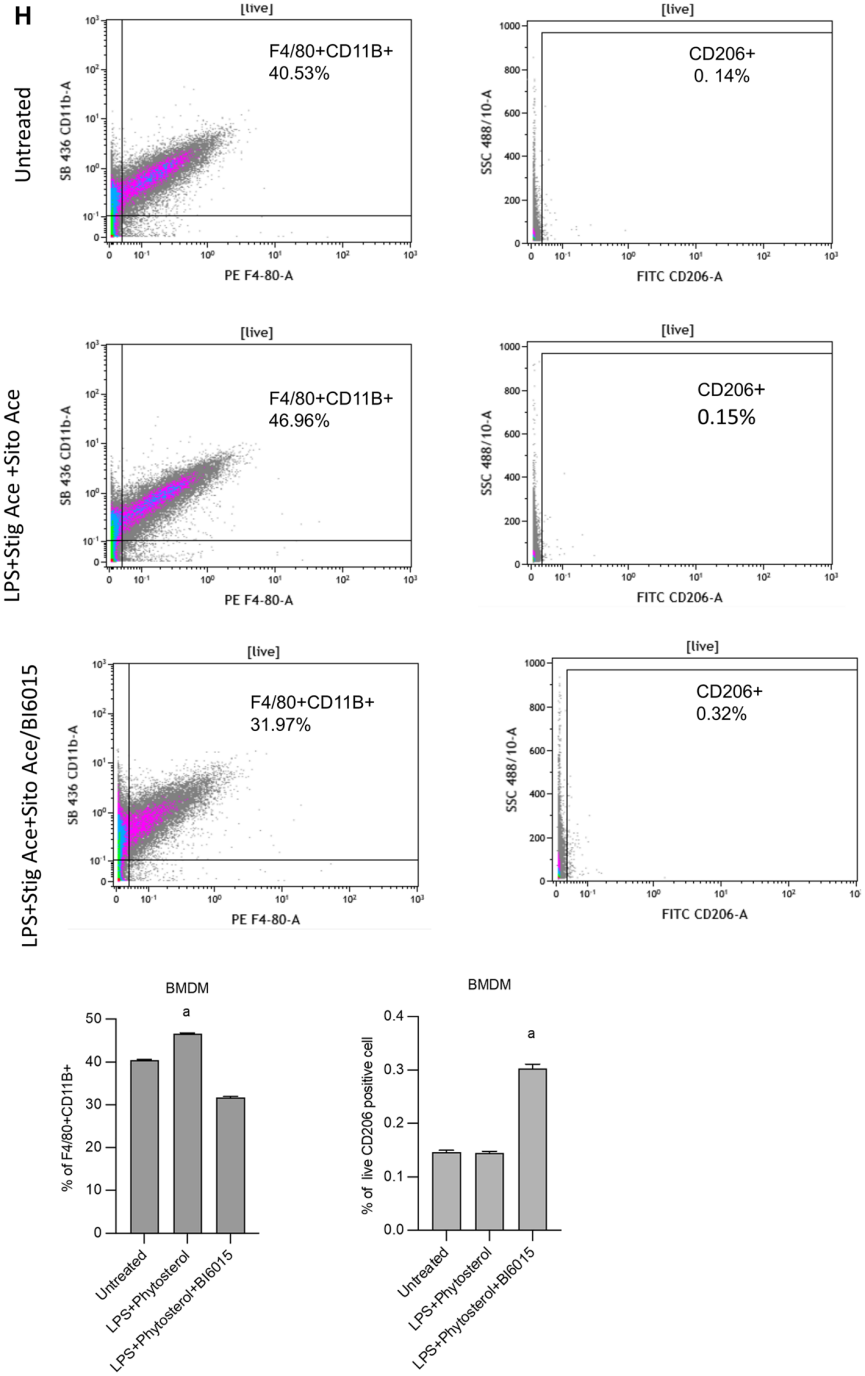
HNF4α antagonist effects on proinflammatory and anti-inflammatory gene expression in hepatic mononuclear cells and BMDMs. (**A**)–(**F**). mRNA expression of liver macrophage markers was measured in cells isolated from Chow, DSS-PN, and DSS-PN/BI6015 mice. Intrahepatic mononuclear cells: (**A**) *Adgre1*(F4/80), data are from 7 Chow, 6 DSS-PN and 4 DSS-PN/BI6015 mice (**B**) *Itgam* (CD11B)*,* data are from 5 Chow, 4 DSS-PN and 4 DSS-PN/BI6015 mice (**C**) *Retnla*, data are from 7 Chow, 5 DSS-PN and 4 DSS-PN/BI6015 mice and (**D**) *Klf4,* data are from 6 Chow, 6 DSS-PN and 4 DSS-PN/BI6015 mice BMDMs: (**E**) *Adgre1,* (F4/80), data are from 4 Chow, 4 DSS-PN and 4 DSS-PN/BI6015 mice and (**F**) *Il-1b*, data are from 4 Chow, 4 DSS-PN and 4 DSS-PN/BI6015 mice*.* (**G**) Liver sections from Chow, DSS-PN, and DSS-PN/BI6015 treated mice were immuno-stained with antibodies against F4/80. Representative images are shown of at least 3 mice per treatment (CV, central vein, PT, portal triad). Quantification by pixels for F4/80 positive cells (200X) shown in bar graph. Data are mean + /− SEM. Data points in (**A**)–(**F**) represent individual mice. (H) BMDM cells were incubated with HNF4α antagonist BI6015 (+ /−) overnight followed by addition of LPS and stig + sito × 4 h, and cells were harvested and flow cytometry analysis was performed for F4/80 + CD11B and Cd206 + cells. Statistical analysis was performed by one-way ANOVA with Tukey’s correction for multiple comparisons. ^a^*p* < 0.0001; ^b^*p* < 0.01 or ^c^*p* < 0.05 versus all other groups.

We have reported a crucial role in the PNAC mouse model for altered intestinal barrier function leading to increased permeability and absorption of LPS into the portal vein, which then activated hepatic macrophages to secrete pro-inflammatory cytokines, particularly IL-1β^10,16^. We also showed that the plant sterols, stigmasterol (stig) and sitosterol (sito) found in soybean oil (SO) PN lipid emulsions, were capable of transcriptional activation of liver macrophages to produce pro-inflammatory cytokines both in vivo in PNAC mice and in vitro in mouse bone marrow derived macrophages (BMDMs)^10,12^. Moreover, hepatic expression of genes representing recruited and inflammatory macrophages (*Adgre1*, *Itgam* [CD11B]) was significantly increased in PNAC mouse liver. Therefore, we next examined the effects of HNF4α antagonism on intrahepatic mononuclear cell (IHMC) expression of the aforementioned genes using qRT-PCR and immunohistochemistry. B16015 treatment in DSS-PN mice significantly reduced the presence of activated macrophages as demonstrated by reduced mRNA expression of *Adgre1* and *Itgam* in hepatic IHMC and BMDM (Fig. 3A,B), and increased the expression of anti-inflammatory genes *Klf4* and *Retnla (*Fig. 3C,D). Furthermore, the increased hepatic transcription in DSS-PN mice of *Il-1b* in BMDM was decreased by BI6015 treatment to levels observed in Chow controls (Fig. 3E,F). The number of hepatic macrophages by immunohistochemistry was similar in all three groups, indicating a qualitative change in macrophage phenotype without quantitative differences. (Fig. 3G). To determine the effect of BI6015 on phenotypic characteristics of macrophages, we performed flow-cytometry analysis for F4/80 + CD11B + positive cells (pro-inflammatory marker) and CD206 + positive (anti-inflammatory marker) in BMDM cells that were incubated with BI6015 overnight followed by exposure to LPS and stig acetate + sito acetate for 4 h. Results showed increased abundance of CD11B positive cells with exposure to LPS and stig + sito which was abrogated by BI6015 pre-treatment. B16015 pre-treatment of BMDM cells was also associated with a phenotypic switch to increased abundance of CD206 positive cells upon exposure to LPS and stig + sito (Fig. 3H).

### Effect of HNF4α antagonism in Raw 264.7 cells and BMDMs

**Figure 4:**
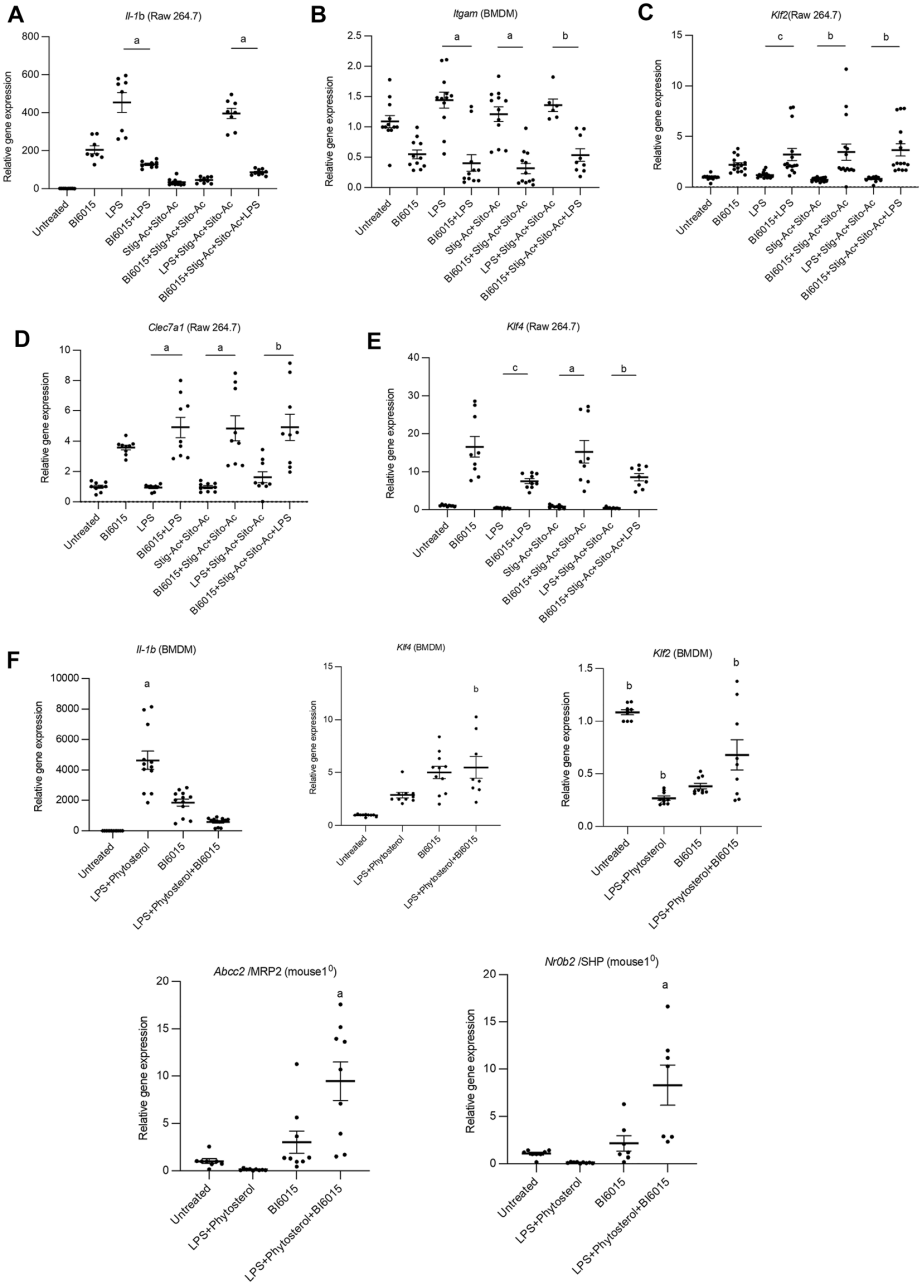
HNF4α antagonist effects on proinflammatory and anti-inflammatory gene expression in Raw 264.7 cells and BMDM and primary mouse hepatocyte gene expression. Cultured wild type mouse BMDMs were incubated with HNF4α antagonist BI6015 overnight followed by addition of LPS or + /− stig + sito × 4 h, and cells were harvested, and mRNA analyzed by qPCR. mRNA expression was determined after normalization to *Hprt1* as an endogenous control gene and expressed relative to results obtained from untreated (**A**) *Il-1b* in Raw264.7 cells*,* (**B**) *Itgam* in BMDMs, (**C**) *Klf2* in Raw264.7 cells, (**D**) *Clec7a1* in Raw264.7 cells, and (**E**) *Klf4* in Raw264.7 cells. (**F**) Co-culture was conducted using mouse BMDM conditioned media transferred to cultured mouse primary hepatocytes and mRNA analyzed by qPCR for *Il-1b, Klf4*, and *Klf2* in BMDMs, and *Abcc2, Nr0b2* in primary hepatocytes. mRNA expression was determined after normalization to *Hprt1* as an endogenous control gene and expressed relative to results obtained from untreated cells. Three independent experiments were presented in (**A**)–(**E**). Data are mean + /− SEM. Statistical analysis was performed by one-way ANOVA with Tukey’s.

Since LPS absorbed from injured intestine and exposure to phytosterols is associated with activation of hepatic macrophages in PNAC^10,16^, we next determined the in vitro effect of BI6015 on pro-inflammatory and anti-inflammatory gene transcription in the Raw 264.7 macrophage cell line and in BMDMs exposed to LPS and phytosterols. Cells were incubated with BI6015 overnight followed by addition of LPS or stig + sito or both for 4 h and *Il-1b*, *Klf2*, *Klf4*, *Clec7a1* and *Itgam* mRNA was measured by qPCR. Transcription in response to LPS and phytosterols was significantly increased in Raw 264.7 cells (*Il-1b)* and BMDMs (*Il-1b* and *Itgam*) which was markedly attenuated by BI6015 pre-treatment (Fig. 4A,B,E). *Itgam* mRNA was not expressed in Raw 264.7 cells in qPCR study. In contrast, B16015 markedly upregulated the mRNA levels of the anti-inflammatory genes *Klf2*, *Clec7a1* and *Klf4 *(Fig. 4C–E), consistent with antagonism of HNF4α inducing a switch from a pro-inflammatory to an anti-inflammatory macrophage phenotype. We next determined the effect of BI6015 treatment of macrophages exposed to LPS and phytosterols on primary mouse hepatocytes exposed to LPS and phytosterols. Cultured wild type mouse BMDMs were incubated with BI6015 + /− overnight followed by addition of LPS and stig + sito x 4 h, media was collected by centrifugation and transferred to cultured mouse primary hepatocytes which were harvested after overnight, and mRNA was analyzed by qPCR in both BMDMs and hepatocytes. BI6015 treatment induced expression of *Klf4* and *Klf2* and suppressed expression of *Il-1b* in BMDMs. *Nr0b2* and *Abcc2* expression was suppressed in hepatocytes exposed to LPS and phytosterols; media from B16015-treated BMDMs promoted marked induction of *Abcc2* and *Nr0b2* mRNA in primary mouse hepatocytes (Fig. 4F).

### BI6015 suppresses IL-1β and phytosterol-dependent activation of NFκB and induces ***Abcc2 and Abcg5***

**Figure 5:**
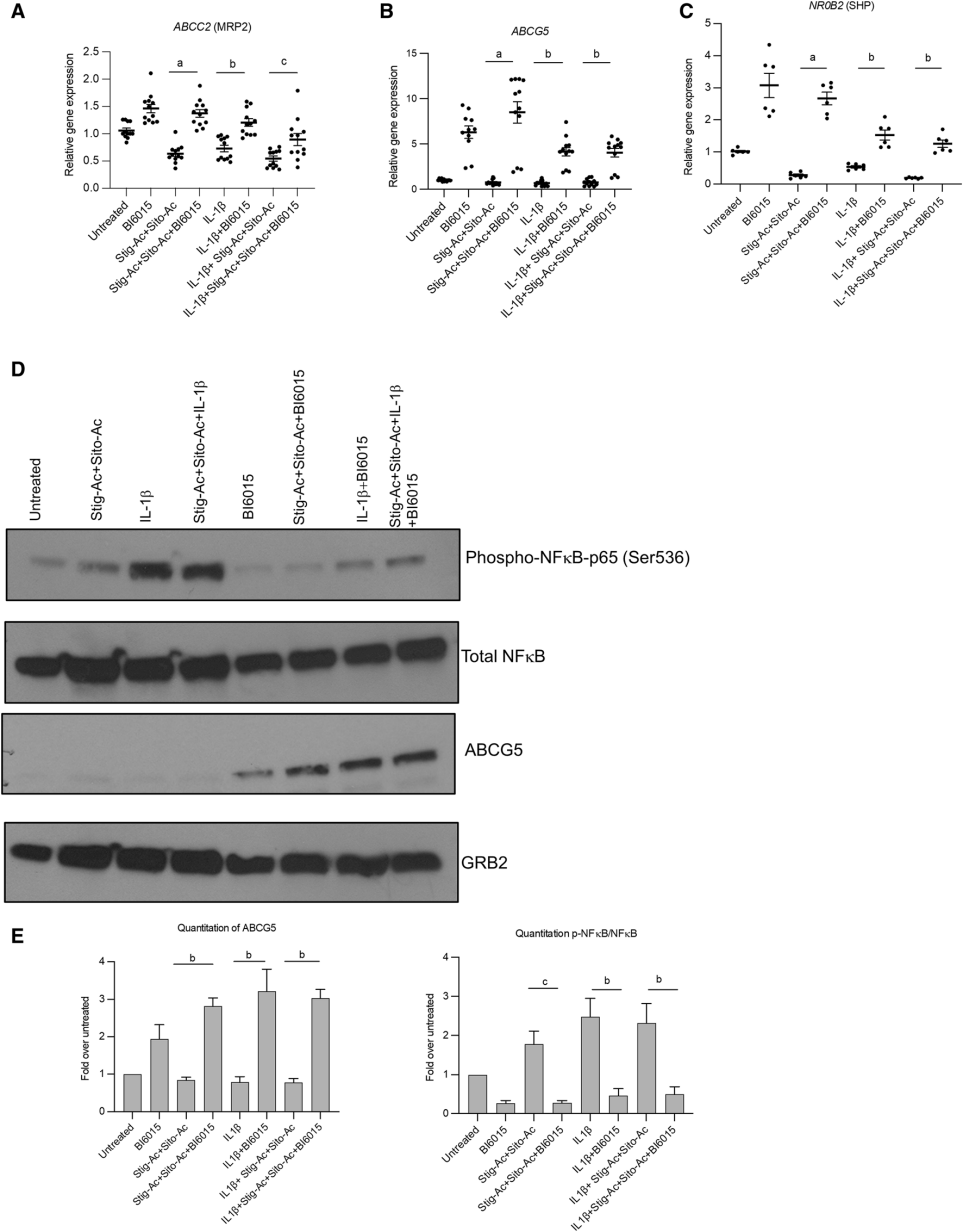

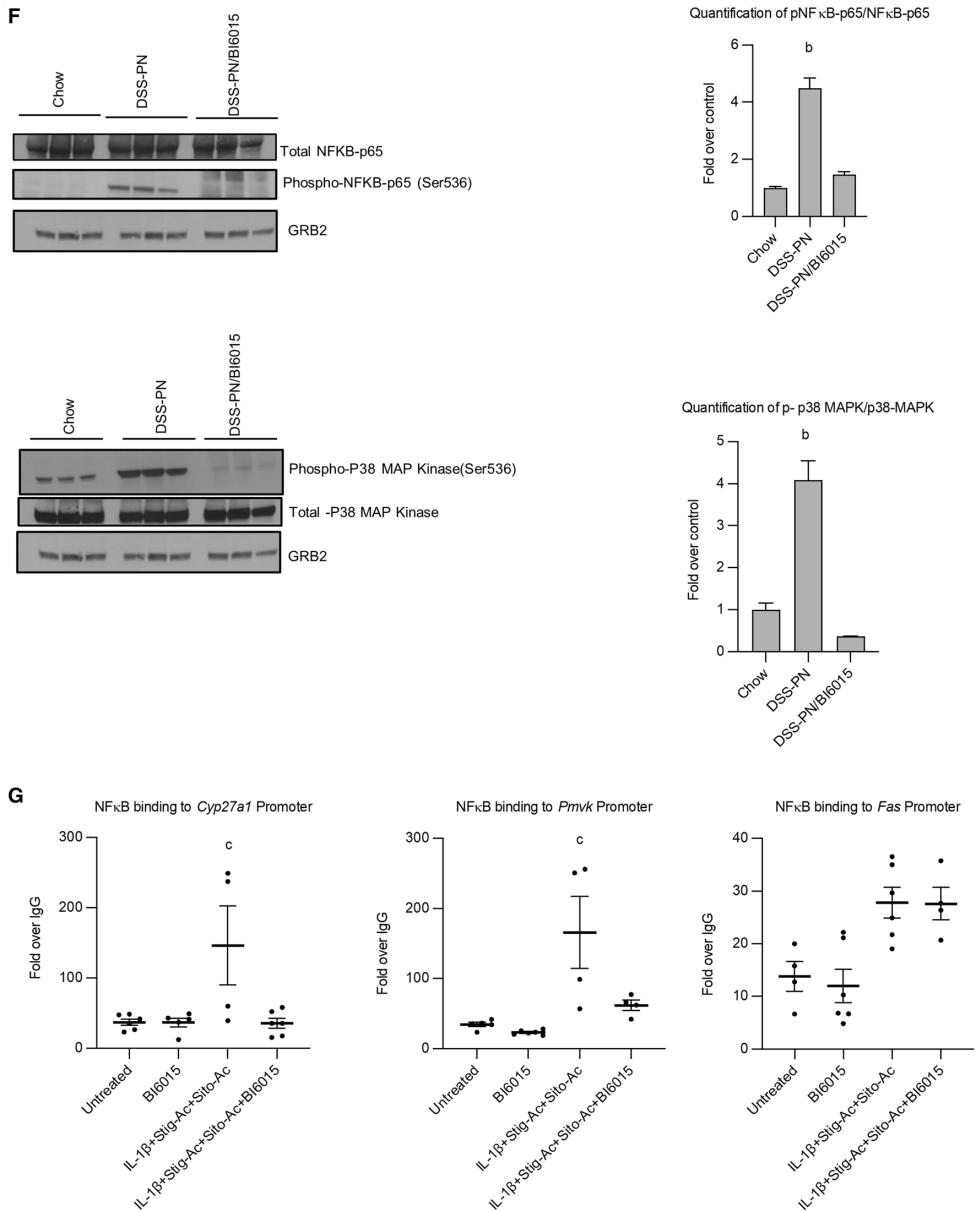

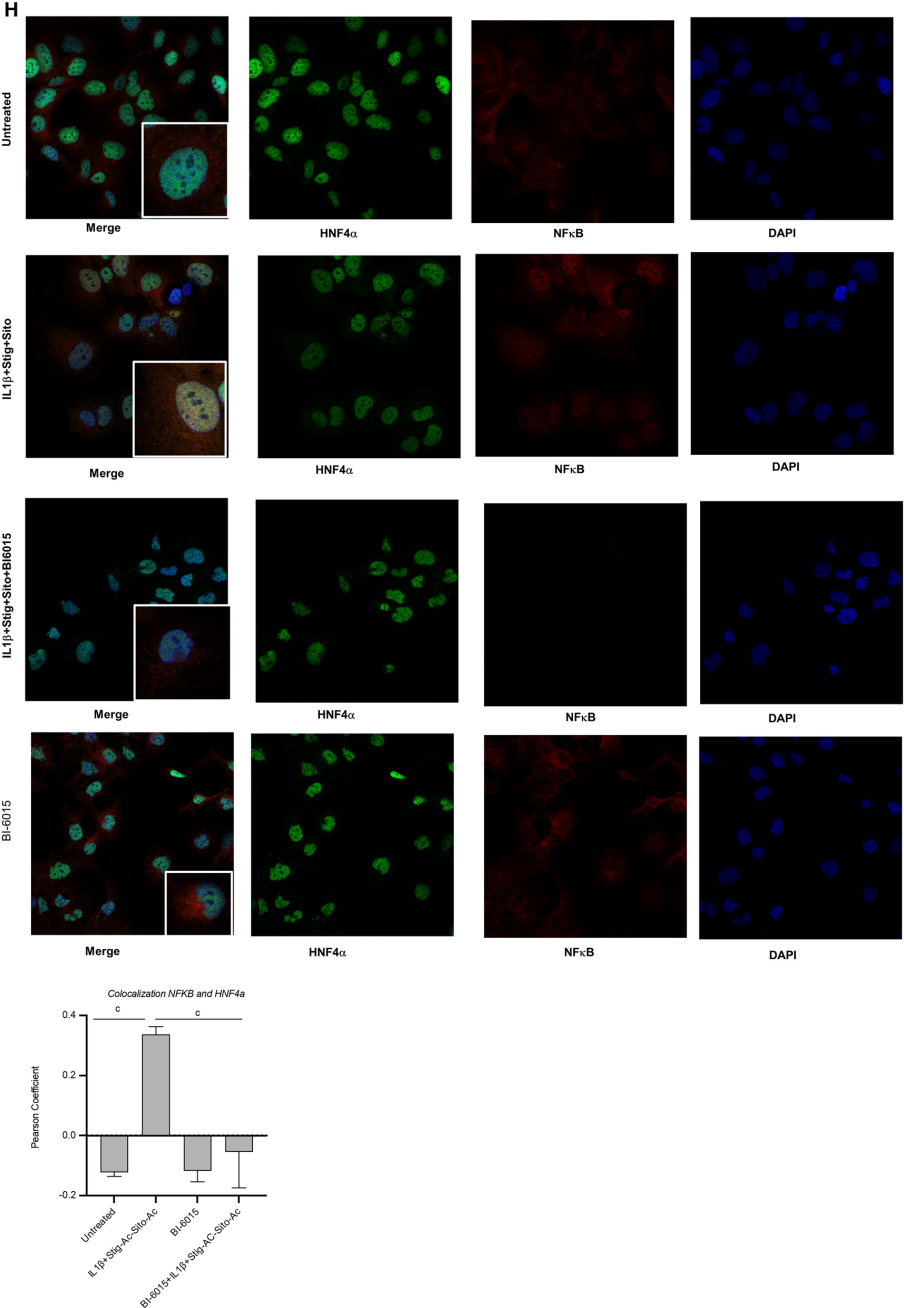
HNF4α antagonist effects on expression of bile and sterol transporters and NFκB activation in HepG2 cells or PNAC mice. HepG2 cells were incubated with HNF4α antagonist BI6015 for 4 h followed by addition of + /− IL-1β or + /− stig + sito overnight, cells were harvested, and mRNA and protein analyzed. mRNA expression was determined after normalization to *Hprt1* as an endogenous control gene and expressed relative to results obtained from untreated (**A**) *ABCC2* mRNA, (**B**) *ABCG5* mRNA, and (**C**) *NR0B2* mRNA. Three independent experiments were presented in (**A**)–(**E**). (**D**) One representative of 3 immunoblots of p NFκB -p65 and ABCG5 protein in lysates extracted from HepG2 cells incubated as described in (**A**). (**E**) Quantification from 3 different blots of ABCG5 and p- NFκB -p65 protein from immunoblots represented in (**D**). (**F**) Immunoblot of isolated hepatocyte homogenates from Chow, DSS-PN and DSS-PN/BI6015 mice for p NFκB -p65 and total NFκB -p65 and IDV normalized to GRB2 and expressed relative to Chow control. Immunoblot of p-p38MAPK and total p38MAPK under same conditions. Data are from 3 Chow, 3 DSS-PN and 3 DSS-PN/BI6015 mice. (**G**) ChIP assay demonstrating NFκB binding to the promoters of *CYP27A*, *PMVK* and *FAS* in cell homogenate from HepG2 cells incubated as described in (**A**). Data presented as fold change over IgG. Each data point represents an individual experiment. (**H**) Confocal microscopy analysis of immunostaining of HNF4α (green), NFκB -p65 (red) and DAPI (blue) in HepG2 cells for experiment described in A showing colocalization of HNF4α and NFκB -p65 in nucleus of IL-1β and phytosterol (stig + sito) treated cells which was prevented by BI6015. One representative of 3 images. Statistical analysis was performed by one-way ANOVA with Tukey’s correction for multiple comparisons. ^a^*p* < 0.0001; ^b^*p* < 0.01 or ^c^*p* < 0.05 for (**A**)–(**E**) and (**H**) between groups, and for (**F**) and (**G**) versus all other groups.

We have previously shown that both macrophage-derived IL-1β and PN-derived phytosterols were important mediators of cholestatic injury in the PNAC mouse model^12,14^. To determine if HNF4α antagonism protected hepatocytes from the suppressive effects of IL-1β and phytosterols on bile and sterol transporter gene expression^10,12,16^, HepG2 cells were pretreated with IL-1β (10 ng/ml × 4h) and phytosterols followed by overnight incubation with 5 µM BI6015. Cells were then harvested and mRNA of *ABCC2, NR0B2 and ABCG5* by qRT-PCR and protein expression of NFκB and ABCG5 were measured. BI6015 increased mRNA expression of *ABCC2* and mRNA and protein expression of *ABCG5* and decreased phosphorylation of NFκB (Fig. 5A–E). We similarly found that BI6015 treatment in vivo in DSS-PN mice suppressed phosphorylation of NFκB-p65 as well as p38 MAP kinase in hepatocytes isolated from mouse liver after 14 days of PN (Fig. 5F). Finally, we determined if HNF4α antagonism could reduce NFκB recruitment to promoters in which there are shared binding sites for these two transcription factors. ChIP assays conducted in HepG2 cells that were pretreated with IL-1β (10 ng/ml) and phytosterols followed by overnight incubation with 5 µM BI6015 showed decreased binding of NFκB to the shared promoters of both *CYP27A and PMVK* in the presence of B16015 (Fig. 5G). In contrast, BI6015 had no effect on NFκB binding to the *Fas* promoter which does not have a shared binding site for HNF4α with NFκB. Furthermore, BI6015 treatment decreased the colocalization in the nucleus, shown by confocal microscopy, of HNF4α and NFκB in HepG2 cells treated with IL-1β + stig + sito (Fig. 5H). Taken together, these data demonstrate that HNF4α antagonism can interfere with NFκB signaling and its role in downregulating bile and sterol transporter expression during PNAC.

## Discussion

The progression of PNAC in patients with IF is characterized by altered intestinal barrier function and dysbiosis, hepatic macrophage activation and cytokine generation leading to NFκB-mediated perturbations of nuclear receptor regulation of hepatocyte canalicular bile and sterol transporters and bile acid synthesis, and the cholestatic toxicity of PN lipid emulsions. Left unchecked, PNAC can progress to biliary cirrhosis, portal hypertension and liver failure that may require liver or multi-visceral transplantation^1,5^ The early events in PNAC have been labeled the cholestatic/inflammatory or the acute/active phase of IFALD in infants and children^6,19–21^. In the current study, we employed a murine model that combined intestinal injury and altered barrier function with PN infusion, that mimics closely the pathophysiology of the cholestatic/inflammatory phase of PNAC in humans^6^, and observed upregulation of HNF4α in the liver during PNAC as well as co-precipitation with NFκB. Antagonism of HNF4α signaling normalized liver biochemistries and hepatocyte bile and sterol transporter and *CYP7A1* expression and reduced hepatic proinflammatory macrophage activation in DSS-PN mice. Moreover, NFκB binding to promoters with shared HNF4α binding sites was significantly reduced by HNF4α antagonism. Taken together, these data suggest that targeting hepatic NFκB through antagonism of HNF4α may be an effective approach to reverse cholestasis and hepatocellular injury in PNAC.

NFκB plays a pivotal role in the pathogenesis of tissue injury in inflammatory and autoimmune hepatic diseases, the progression to fibrosis and ultimately the development of hepatocellular carcinoma (HCC), thus it may represent a target for the prevention or treatment of these conditions^22^. However, NFκB also has important beneficial biological functions such as the development of immune cell and lymphoid organs, immune homeostasis, and immune responses^23–25^. If NFκB is to be examined as a therapeutic target in liver diseases, interventions that employ either pathway-specific NFκB blockade or a moderate effect on NFκB activity will be essential to avoid the increase in hepatic injury associated with complete NFκB blockade^26^. In the current study, we identified by in silico analysis that NFκB and HNF4α shared binding sites on gene promoters and therefore we hypothesized that interrupting HNF4α signaling could be a strategy for mitigating the negative effects of NFκB activation in liver^16,27,28^. We showed that NFκB and HNF4α co-localize in the nucleus of HepG2 cells that are exposed to IL-1β and phytosterols, the two most important factors mediating PNAC. IL-1β has been shown to induce translocation of NFκB to the nucleus, where it upregulates target proinflammatory genes and downregulates genes involved in bile acid transport. Interestingly, treatment with the HNF4α antagonist BI6015 prevented both the phosphorylation and translocation to the nucleus of NFκB, which was associated with protection against PNAC. Thus, interrupting HNF4a signaling reduced NFκB binding and its subsequent effects on gene expression in cells exposed to the two factors most implicated in the pathogenesis of PNAC, IL-1β and phytosterols. NFκB and HNF4α both are regulated by p38 MAP kinase that was induced in PNAC mice but inhibited in BI6015 treated mice^29,30^, suggesting a possible feedback loop from HNF4α to p38MAPKinase.

Recent human studies evaluating gene expression and histology of livers from infants with IF and PNAC confirmed the hepatic gene expression and biochemical findings in our PNAC mouse model^10–12,16,21,31^. This includes increased hepatic and serum accumulation of phytosterols^12,21^, hepatic macrophage infiltration^10^, increased hepatic inflammatory cytokine expression in conjunction with transcriptional suppression of canalicular sterol (*ABCG5/8*), bile acid (BSEP) and bilirubin (MRP2) transporters^31^. We have previously reported that modulation of transcription factors FXR and LRH-1 could mitigate cholestasis in the PNAC mouse model^16^. Here, we extend those findings in the PNAC mouse to show that pharmacologic antagonism of another major hepatic transcription factor, HNF4α, similarly led to upregulation of FXR and LRH-1 and expression of downstream bile acid exporters and sterol transporters^16,18,32^ with prevention of PNAC. The significantly suppressed phosphorylation of NFκB-p65, which is required for the development of PNAC in the mouse model, and its reduced binding to BSEP and LRH-1 promoters, likely contributed to the reversal of suppression of BSEP, LRH-1 and *Abcg5/8* at the mRNA level, resulting in improved export of bile acids and phytosterols across the canalicular membrane^33,34^.

In HepG2 cells exposed in vitro to IL-1β and phytosterols, antagonism of HNF4a similarly suppressed NFκB activation, which was associated with increased transcription of *ABCG5, ABCC2* and SHP. In the mouse PNAC model, antagonism of HNF4a decreased NFκB binding to the *Abcb11* promoter, normalized *Abcb11* expression and attenuated cholestasis. Previously we have shown that in vitro pharmacological inhibition of NFκB increased the expression of *Nr1h4*, *Abcb11*, *Nr0b, Abcc2, and Abcg5/8*^10^. Taken together, these results indicate the role of NFκB in mediating differential gene expression of bile and sterol transporters and support NFκB as a target for mitigation of PNAC.

We next examined the effect of HNF4a inhibition on the role of macrophages in the PNAC mouse model and in cultured macrophages. In the PNAC mouse model and in infants with PNAC, hyperplasia and hypertrophy of hepatic macrophages is associated with progressive cholestasis and initiation of fibrosis^31,35^. Moreover, intestinal LPS and phytosterols promoted hepatic macrophage activation in vivo and directly induced transcription of *Il-1b* in cultured Raw macrophages, thus contributing to the hepatic inflammatory milieu in this mouse model^10,12,16^. Phytosterols have also been suggested to directly promote cholestasis through antagonizing FXR activation of target genes, resulting in reduced *Abcb11*expression^16,36^. In the current study, we show that HNF4α antagonism reduced proinflammatory gene expression and induced expression of anti-inflammatory genes in hepatic and bone marrow macrophages derived from the PNAC mouse. In addition, in vitro HNF4α antagonism similarly suppressed LPS and phytosterol-induced expression of *IL-1b* and *Itgam* and increased the expression of *Klf2* and *Klf4*, which may be associated with resolution of inflammation^37^. Taken together, these data indicate that an additional mechanism for the amelioration of cholestasis by HNF4α antagonism may be through induction of a polarization switch in hepatic macrophages from a pro- to an anti-inflammatory phenotype^16,18,31^.

**Figure 6:**
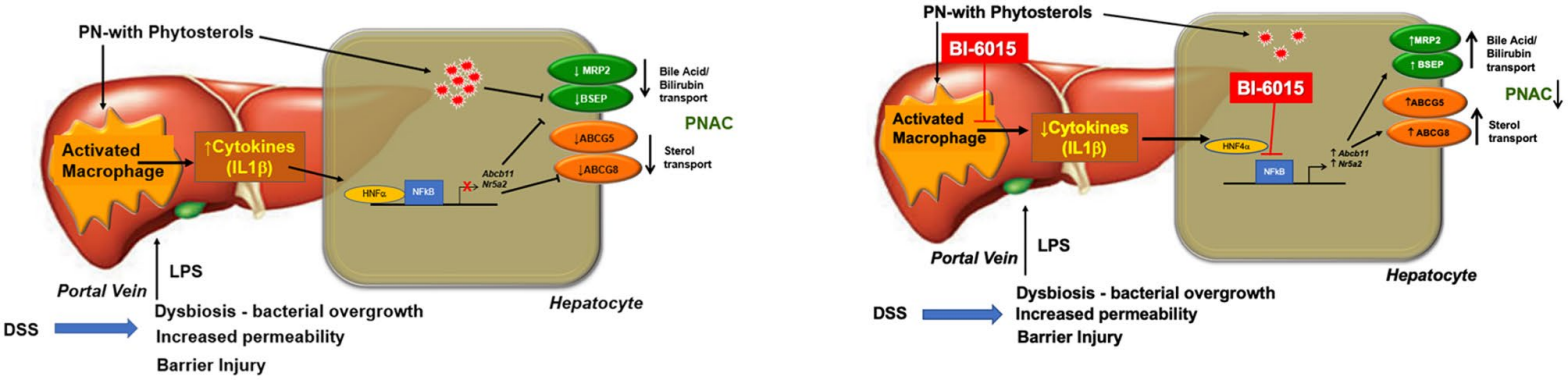
Proposed role of HNF4α in PNAC pathogenesis. Intestinal injury, dysbiosis and hyperpermeability caused by DSS in mouse model, and intestinal failure in humans, promote LPS (and other microbial associated molecular patterns) absorption into portal vein, subsequently recruiting and activating liver macrophages to generate IL-1β which binds to IL-1 receptor on hepatocytes. Phytosterols contained in the PN lipid emulsions may also directly activate hepatic macrophages. This triggers NFκB interactions in the hepatocyte with HNF4α and binding to promoters of bile acid and sterol transporters, with suppression of transcription of these genes. HNF4α antagonism (by BI6015) suppresses activation and binding of NFκB to gene promoters, thus upregulating canalicular *Abcg5/8*, *Abcb11*/BSEP and *Abcc2*/MRP2. This leads to increased hepatocyte secretion and reduces intracellular concentrations of phytosterols, bile acids and conjugated bilirubin, mitigating cholestatic liver injury. In addition, BI6015 induces a transcriptional change in hepatic macrophages from a pro- to anti-inflammatory phenotype that may reduce the inflammatory cascade in PNAC.

In conclusion, this study has shown that HNF4α is upregulated in the murine model of PNAC and that the HNF4α antagonist BI6015 attenuates PNAC by suppressing NFκB activation and signaling while inducing hepatocyte FXR and LRH-1 and their downstream bile and sterol transporters, and by promoting a switch in hepatic macrophage polarization from a pro-inflammatory to anti-inflammatory phenotype. These data identify HNF4α antagonism as a potential therapeutic target for prevention and treatment of PNAC (Fig. 6).

## Methods

### PNAC mouse model

All animal care and experimental procedures were approved by the University of Colorado Anschutz Medical Campus Institutional Animal Care and Use Committee and were carried out according to rules and procedures of US Public Health Service’s Policy for use of laboratory animals and guide for care and use of animals. The Study was carried out following ARRIVE guidelines. The PNAC mouse model was employed as previously described^10,11,16,18^. In brief, C57BL/6 wild-type adult male mice (8 weeks old, 25 g body weight) (Jackson Laboratories, Bar Harbor, ME) were housed in pathogen-free conditions in individual metabolic cages. Male mice were used for our experiments to avoid the effects of female hormones, specifically estrogens, on inhibition of nuclear receptor gene expression, bile formation and bile flow which could confound the results of these experiments in a mouse model of cholestasis^38,39^. To stimulate mild intestinal injury and increased permeability, mice were exposed to 2.0% DSS in the drinking water for 4 days during which time they had access to water and chow ad libitum. After DSS pre-treatment, PNAC mice underwent surgical placement of a central venous catheter (CVC) (Silastic tubing, 0.012 inches internal diameter; Dow Corning, USA) under pentobarbital anesthesia into the right jugular vein with the proximal end tunneled subcutaneously exiting between the shoulder plates. Mice were positioned in a rubber mouse harness (Instech Laboratories, Plymouth Meeting, PA) and the CVC was inserted through a swivel apparatus and attached to an infusion pump (Harvard Apparatus, Holliston, MA). Mice recovered from surgery for 24 h with i.v. normal saline (NS) infused at a rate of 0.23 ml/hr while given *ad lib* access to chow and water. After 24 h, chow was removed, access to water was continued and mice were infused for 14 days with continuous PN (DSS-PN mice) at a rate of 0.29 ml/hr providing a caloric intake of 8.4 kcal/day and SO lipid emulsion (Intralipid^R^, Fresenius, Germany) dose of 5 g/kg/day. Mice were randomly allotted to several groups as follows: (1) Chow mice were age-matched and given free access to chow and water for 14 days without DSS or CVC placement. (2) DSS-PN mice as described above. (3) DSS pretreatment followed by PN for 14 days with the addition of the HNF4α antagonist, BI6015 (Cat No:4641; Tocris, Minneapolis, MN, USA), at dose of 20 mg/kg/body weight per day formulated in DMSO and mixed into the PN solution on days 4 to 14 of PN (DSS-PN/BI6015). On the day of sacrifice, mice were anesthetized with i.p. pentobarbital and blood collected from the retro-orbital plexus and liver removed and divided with one portion placed in formalin for liver histology and a second portion snap frozen in liquid nitrogen and subsequently stored at -80 degrees Celsius until analyzed. Serum aspartate aminotransferase (AST) and alanine aminotransferase (ALT) were analyzed at the University of Colorado Hospital Clinical Chemistry Laboratory from coded serum samples. Total serum bile acids were analyzed in coded specimens using a total bile acid detection kit (Cat No DZ042A, Diazyme Laboratories, Poway, CA) according to the manufacturer’s instructions and total serum bilirubin was measured in coded samples by bilirubin colorimetric kit (Cat No K553, Biovision Inc, Milpitas,CA). To ensure rigor and reproducibility, all samples were analyzed in a blinded manner in coded specimens.

### Hepatic mononuclear cell isolation

Mononuclear cells were isolated from mouse liver, as previously described^10,18^. Briefly, livers were chopped in cold Hanks buffer (Gibco/Invitrogen, Carlsbad, CA) and incubated in Liberase^R^ (Cat No 05989132001 Hoffman La Roche, Germany) and DNAse (Sigma-Aldrich, St. Louis, MO) for 30 min at 37 °C followed by filtration over a 100 µm cell strainer and spun at low speed centrifugation at 25 g for 3 min followed by further filtrations over a 70 µm filter and spun at 300 g for 5 min. Cells were then separated over a 40% Histodenz gradient at 1500 g for 20 min. The cells collected from the gradient, which are composed of primarily macrophages, are referred to as IHMCs in this study and used for RNA isolation.

### RNA isolation and quantitative gene expression analysis

Liver or cellular RNA was extracted using TRIzol^®^ (Invitrogen, Carlsbad, CA, USA) according to manufacturer’s instructions. RNA samples were used for cDNA preparation using a kit (Cat: 1708891; BioRad, Hercules, CA) followed by real-time qRT-PCR using TaqMan probes (Thermo Fisher Scientific, Waltham, MA, USA), as previously described^10,16^.

### Immunoblot and co-immunoprecipitation analysis

Total cell lysates and nuclear fractions were isolated using M-PER™ and NE-PER™ Extraction Reagents (Thermo Fisher Scientific, Waltham, MA, USA) according to the manufacturer’s instructions. Proteins were quantified by BCA Assay (Thermo Fisher Scientific) and separated on 4–20% SDS polyacrylamide gels (Biorad, Hercules, CA) and transferred onto PVDF membranes (EMD Millipore, Billerica, MA, USA,). Membranes were then incubated for 1 h in blocking buffer (Tris-buffered saline, 0.1% Tween; TBS-T), 5% nonfat dry milk and incubated overnight at 4 °C with indicated primary antibodies followed by 1 h incubation with indicated secondary antibodies (Supplementary Table 1). Blots were developed using enhanced chemiluminescence (ECL, Santa Cruz Biotechnologies, Santa Cruz, CA) per the manufacturer’s protocol^16^. For co-immunoprecipitation assay, we used Dynabeads Protein A immunoprecipitation kit (Thermo Fisher Scientific, Waltham, MA, USA) according to the manufacturer’s instructions.

### Isolation of mouse hepatocytes

Mouse hepatocytes were isolated from fresh liver tissue as previously described^16,40^, with the following minor modifications. Briefly, mouse liver was perfused in situ through a 24G catheter inserted into the inferior vena cava using sequential buffers, EBSS containing EGTA and HEPES until liver became opaque (Cat No: 14155 Gibco, Lafayette, CO, USA), liver perfusion media for 6 min (Cat No: 17701 Gibco) and liver digest media containing collagenase for 15 min (Cat No: 17703 Gibco) respectively, at 37 °C. Following perfusion, liver was extracted and filtered using 70 µM strainer and washed with William’s E media (Cat No: 32551, Gibco, Lafayette, CO, USA) plus 10% high-performance, ultra-endotoxin low FBS (ThermoFisher, Waltham, MA, USA) and 1% penicillin and streptomycin (ThermoFisher). After centrifugation at 25 g for 5 min, the hepatocytes were collected for RNA isolation.

### Chromatin immunoprecipitation (ChIP)

ChIP was performed on liver samples that were immediately flash frozen in liquid nitrogen before processing, using a specific antibody to NFκB (cat# 8242s, Cell signaling, San Diego, CA). Amplification of NFκB-p65 binding to the *Nr5a2* and *Abcb11*(mouse) and *Cyp27a1, PMVK* and *Fas* (human) promoter sequences was performed using a specific primer set and subsequent PCR, as previously described^10,16^. ChIP assays were performed using EZ ChIP/Magna ChIP G Kit (Cat No 17-611 EMD/Millipore; Billerica, MA, USA) according to the manufacturer’s instructions.

### Histological analysis

After sacrifice, liver tissues were removed, formalin-fixed, paraffin-embedded and sectioned at 5 microns. Slides were stained immunohistochemically using the pan macrophage marker F4/80 (Cat no MF48000; Invitrogen, CA) at a dilution of 1:400. Slides were treated with DAKO Target Retrieval Solution (Cat No GV805, Agilent technology, Santa Clara, CA) for antigen retrieval technique in a 110 °C Pressure cooker for 50 min as per as per manufacturer’s instructions (DAKO) by University of Colorado Denver Cancer Center Research Histology core..

### Flow cytometry

Wild type mouse bone derived macrophages (BMDM) were pre-treated with BI6015 overnight before adding 10 µM stig and sito (acetate) and LPS (100 ng/ml) for 4 h on the next morning. Then cells were washed and resuspended in staining buffer (Thermo Fisher Scientific, Waltham, MA, USA) and stained with specific antibodies, as previously described^10^. Primary antibodies were used to label cells for F4/80, CD11B and CD206 (Thermo Fisher Scientific, Waltham, MA, USA). Flow-assisted cell analysis was conducted at the University of Colorado Cancer Center Flow Cytometry Core Facility and was performed on Yeti flow cytometry device (Bio-rad,Hercules,CA) and data were analyzed by Kaluza software (Beckman Coulter, Brea,CA).

### Immunostaining

Cells cultured on glass coverslips were fixed with 4% paraformaldehyde and washed with Tris-buffered saline (TBS). All reagents were diluted in TBS, and coverslips were washed by immersion into TBS between each staining step. Coverslips were incubated sequentially with 0.5% Triton-X100 (Sigma,St Louis,MO), blocking buffer (15 min), primary antibody diluted in blocking buffer (overnight, 4 °C) and secondary antibody diluted in blocking buffer (30 h, room temperature). Coverslips were mounted on to glass slides (VWR) using DAPI containing ProLong™ Gold Antifade Mountant with DAPI mounting media (Catalog No: P36931; Thermofisher, Watham, MA) as per manufacturer’s instructions and confocal microscopy was done in Zeiss LSM780 spectral microscope in University of Colorado Denver Anschutz campus at Advanced light Microscopy core.

### Cultured hepatocyte and macrophage experiments

HepG2 cells were treated with 10 ng/ml recombinant murine IL-1β (BD Biosciences, San Jose, CA, USA) or vehicle for 4 h followed by 5 µM BI6015 (Tocris, Minneapolis, MN, USA), an HNF4α antagonist, or 10 µM stigmasterol (stig) acetate and 10 µM sitosterol (sito) acetate (Steraloids, Newport, RI, USA) overnight in DMEM media with transfection agent (Dharmafect, Dharmacon, Lafayette, CO), as described in^10,16^. Cultured Raw 264.7 cells or wild type mouse BMDMs were incubated with HNF4α antagonist BI6015 overnight followed by addition of + /- LPS(100 ng/ml) or + /- stig + sito (10 µM) × 4 h, and cells were harvested, and mRNA analyzed by qPCR.

Cultured wild type mouse BMDMs were incubated with HNF4α antagonist BI6015 overnight followed by addition of LPS (200 ng/ml) and stig + sito(10 µM) × 4 h, and macrophage conditioned media was collected by centrifugation and transferred to cultured mouse primary hepatocytes for overnight incubation; cells were then harvested and mRNA analyzed by qPCR.

### Statistical analysis

qPCR assays were determined in triplicate for each mouse and the average from each mouse was used to calculate the mean ± standard error of mean for each treatment group. For cell culture experiments, gene expression was determined in triplicate, and results from three representative experiment are shown. To ensure rigor, animals were randomly allocated to treatment groups and all samples were coded and were analyzed blinded to the treatment group. The number of mice in each experimental group was 3–7 and is provided in the text, figures, and/or legends. One-way analysis of variance and Tukey’s correction for multiple comparisons were used to determine statistical significance for multiple group comparisons. The level of significance between two groups was determined by Student’s 2-tailed unpaired *t*-test. P-value < 0.05 was considered statistically significant. The statistical analysis was done in PRISM Graph Pad software (La Jolla, CA, USA)^16^. All authors had access to the study data and had reviewed and approved the final manuscript.

## Supplementary Information

Supplementary Information.

Supplementary Table 1.

## Data Availability

The datasets used and/or analyzed during the current study are available from the corresponding author on reasonable request.

## Abbreviations

ABCB11: ATP-binding cassette, sub-family B member 11
ABCC2: ATP-binding cassette sub-family C member 2
BSEP: Bile salt export pump
DSS: Dextran sulfate sodium
HNF4a: Hepatocyte nuclear factor 4 alpha
IFALD: Intestinal failure associated liver disease
NFκB: Nuclear factor kappa-light-chain-enhancer of activated B cells
NR5A2: Nuclear receptor subfamily 5, group A member 2
MRP2: Multidrug resistance-associated protein 2
NR0B2: Nuclear receptor subfamily 0, group B member 2
SHP: Small heterodimer partner
PN: Parenteral nutrition
PNAC: Parenteral nutrition associated cholestasis
stig: Stigmasterol
sito: Sitosterol
TPN: Total parental nutrition
