# Publisher Correction: Pharmacologic inhibition of HNF4α prevents parenteral nutrition associated cholestasis in mice

**DOI:** 10.1038/s41598-023-38001-3

**Published:** 2023-07-19

**Authors:** Swati Ghosh, Michael W. Devereaux, David J. Orlicky, Ronald J. Sokol

**Affiliations:** 1 Section of Pediatric Gastroenterology, Hepatology and Nutrition, Department of Pediatrics, Pediatric Liver Center, Digestive Health Institute, Children’s Hospital Colorado, University of Colorado School of Medicine, 13123 E. 16th Ave, Aurora, CO 80045 USA; 2 Department of Pathology, University of Colorado School of Medicine, 12801, E 17th Ave, Aurora, CO 80045 USA

Correction to: *Scientific Reports* 10.1038/s41598-023-33994-3, published online 12 May 2023

The original version of this Article contained an error in Figure 5H, where the text on the left side of the panels did not display correctly. In addition, the histogram graph that followed the confocal microscopy images was omitted.

The original Figure 5 and accompanying legend appear below.

**Figure 5:**
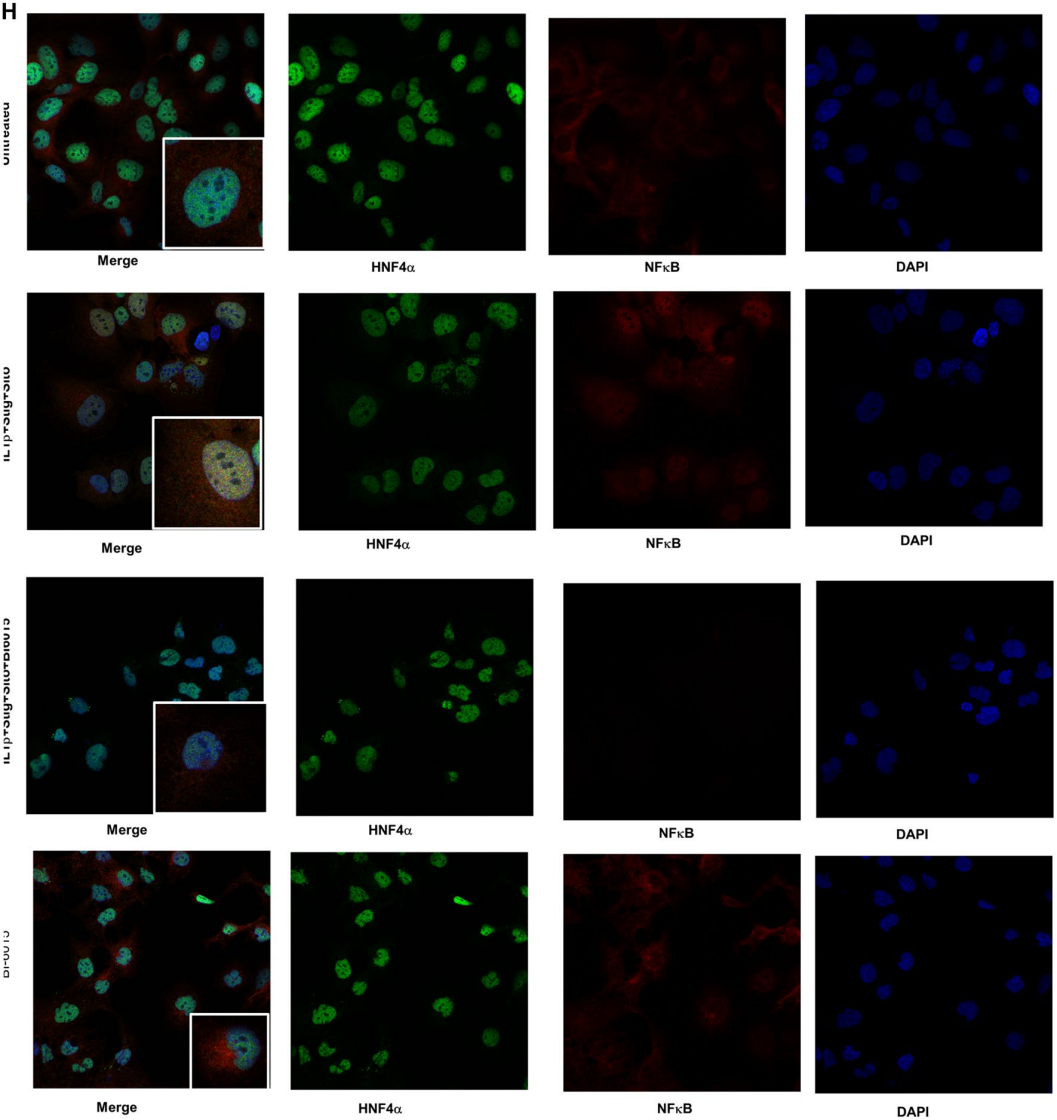
HNF4α antagonist effects on expression of bile and sterol transporters and NFκB activation in HepG2 cells or PNAC mice. HepG2 cells were incubated with HNF4α antagonist BI6015 for 4 h followed by addition of + /− IL-1β or + /− stig + sito overnight, cells were harvested, and mRNA and protein analyzed. mRNA expression was determined after normalization to *Hprt1* as an endogenous control gene and expressed relative to results obtained from untreated (**A**) *ABCC2* mRNA, (**B**) *ABCG5* mRNA, and (**C**) *NR0B2* mRNA. Three independent experiments were presented in (**A**)–(**E**). (**D**) One representative of 3 immunoblots of p NFκB -p65 and ABCG5 protein in lysates extracted from HepG2 cells incubated as described in (**A**). (**E**) Quantification from 3 different blots of ABCG5 and p- NFκB -p65 protein from immunoblots represented in (**D**). (**F**) Immunoblot of isolated hepatocyte homogenates from Chow, DSS-PN and DSS-PN/BI6015 mice for p NFκB -p65 and total NFκB -p65 and IDV normalized to GRB2 and expressed relative to Chow control. Immunoblot of p-p38MAPK and total p38MAPK under same conditions. Data are from 3 Chow, 3 DSS-PN and 3 DSS-PN/BI6015 mice. (**G**) ChIP assay demonstrating NFκB binding to the promoters of *CYP27A*, *PMVK* and *FAS* in cell homogenate from HepG2 cells incubated as described in (**A**). Data presented as fold change over IgG. Each data point represents an individual experiment. (**H**) Confocal microscopy analysis of immunostaining of HNF4α (green), NFκB -p65 (red) and DAPI (blue) in HepG2 cells for experiment described in A showing colocalization of HNF4α and NFκB -p65 in nucleus of IL-1β and phytosterol (stig + sito) treated cells which was prevented by BI6015. One representative of 3 images. Statistical analysis was performed by one-way ANOVA with Tukey’s correction for multiple comparisons. ^a^*p* < 0.0001; ^b^*p* < 0.01 or ^c^*p* < 0.05 for (**A**)–(**E**) and (**H**) between groups, and for (**F**) and (**G**) versus all other groups.

The original Article has been corrected.

