# Supplementary figures and images for: Pharmacologic inhibition of HNF4α prevents parenteral nutrition associated cholestasis in mice
Source: Sci Rep. 2023 May 12;13:7752. doi: 10.1038/s41598-023-33994-3 (PMC10182080; doi:10.1038/s41598-023-33994-3)

Figure 1B

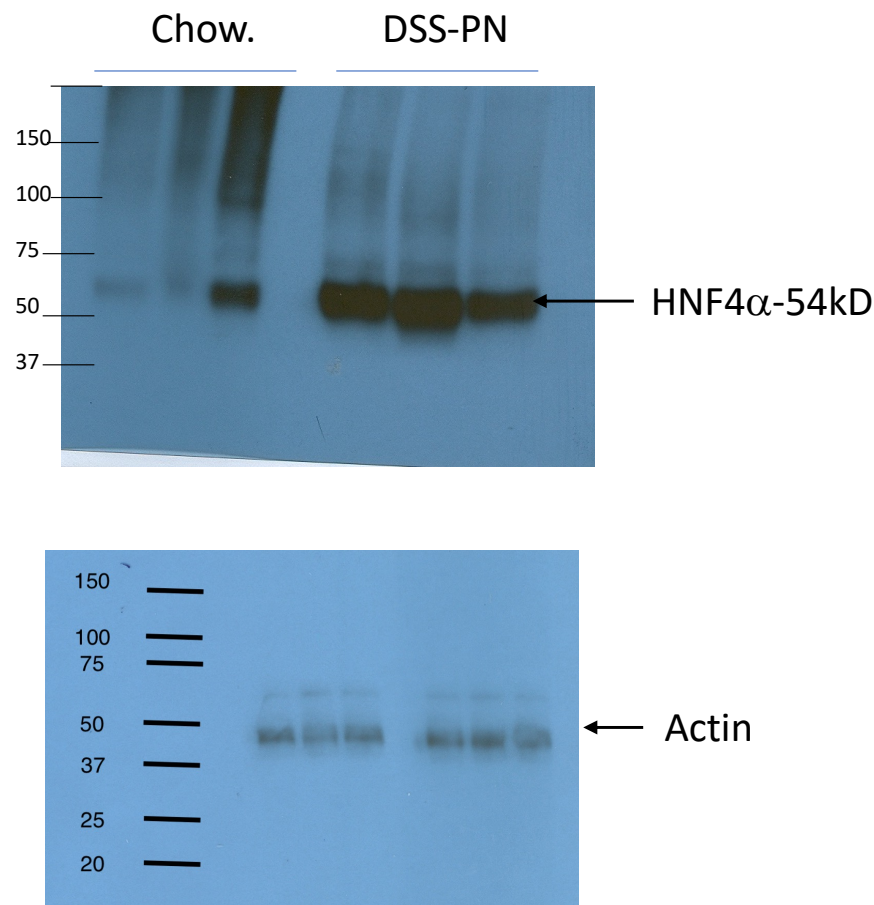

Figure 1D

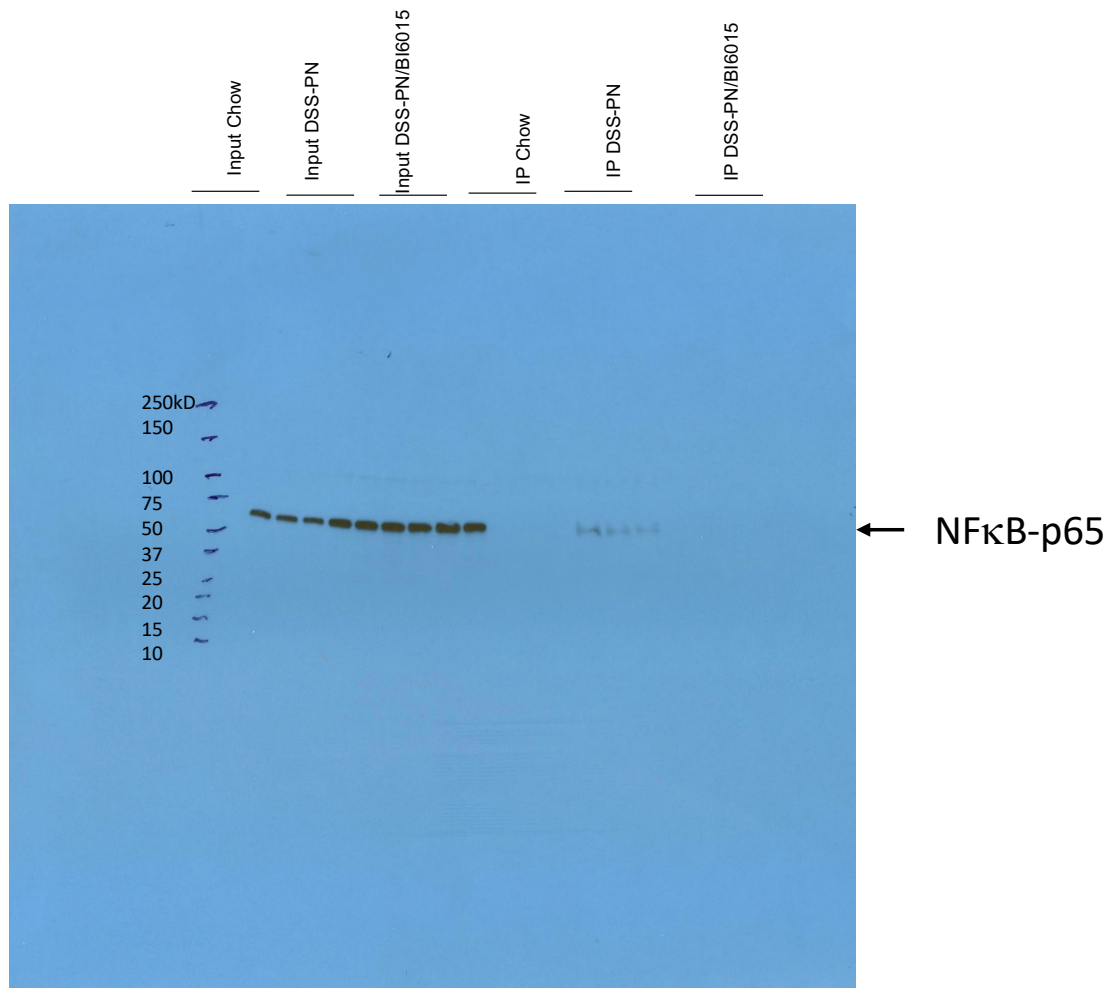

Figure 1D

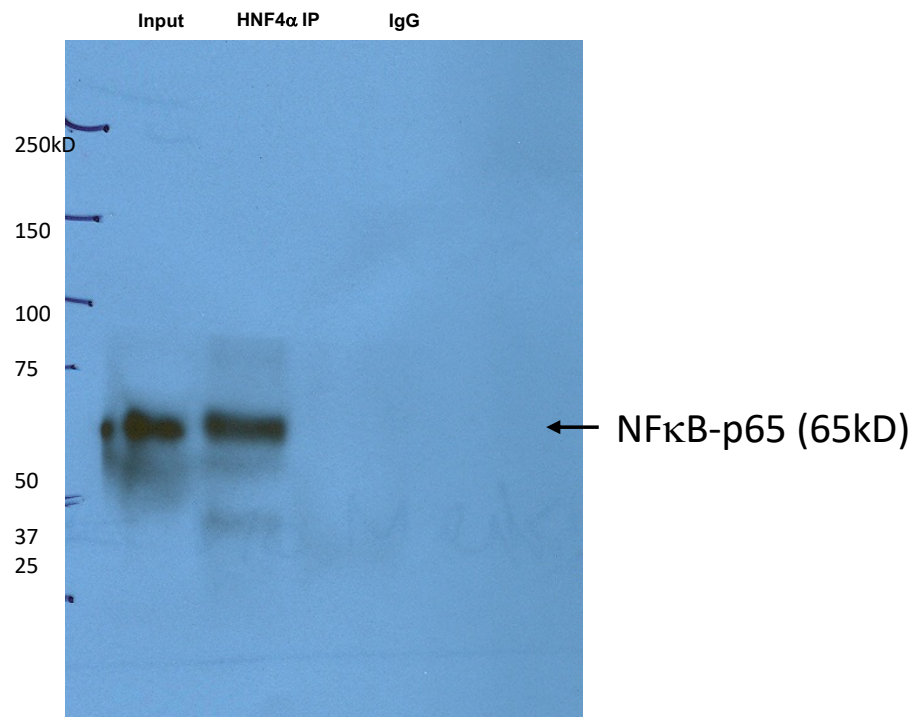

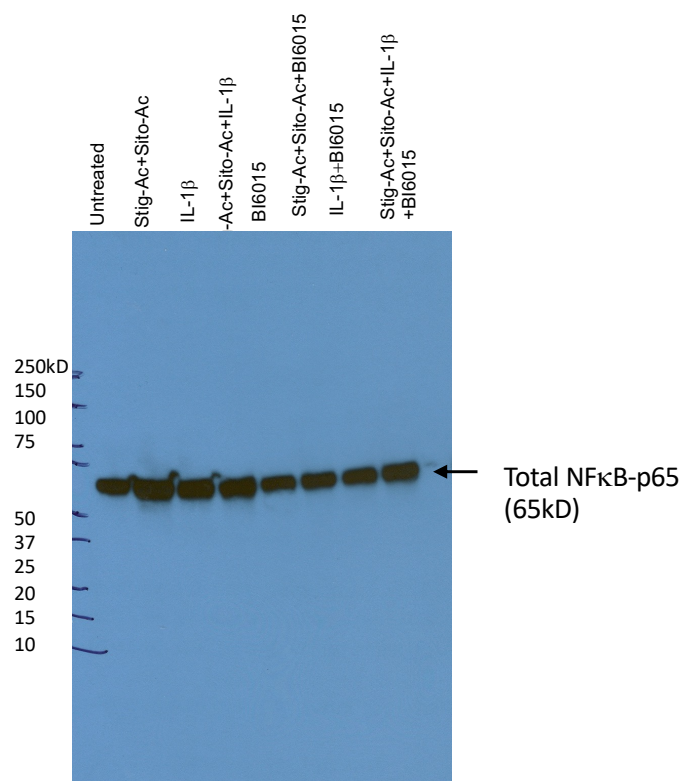

Figure 5D

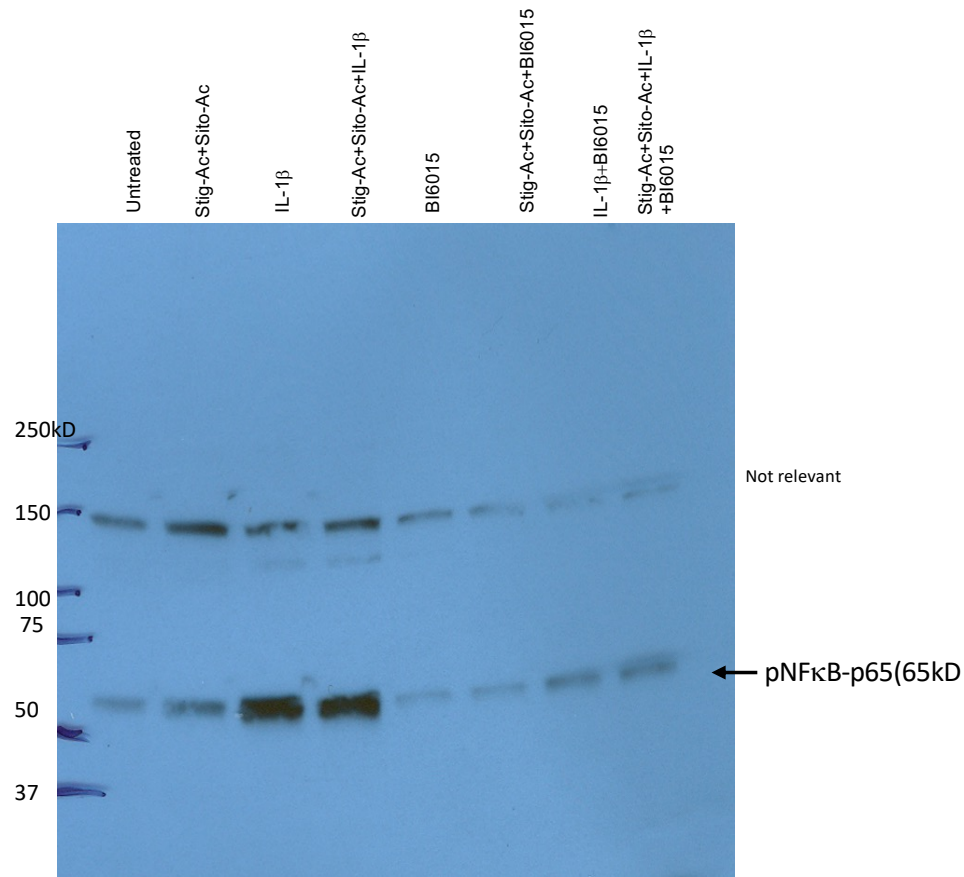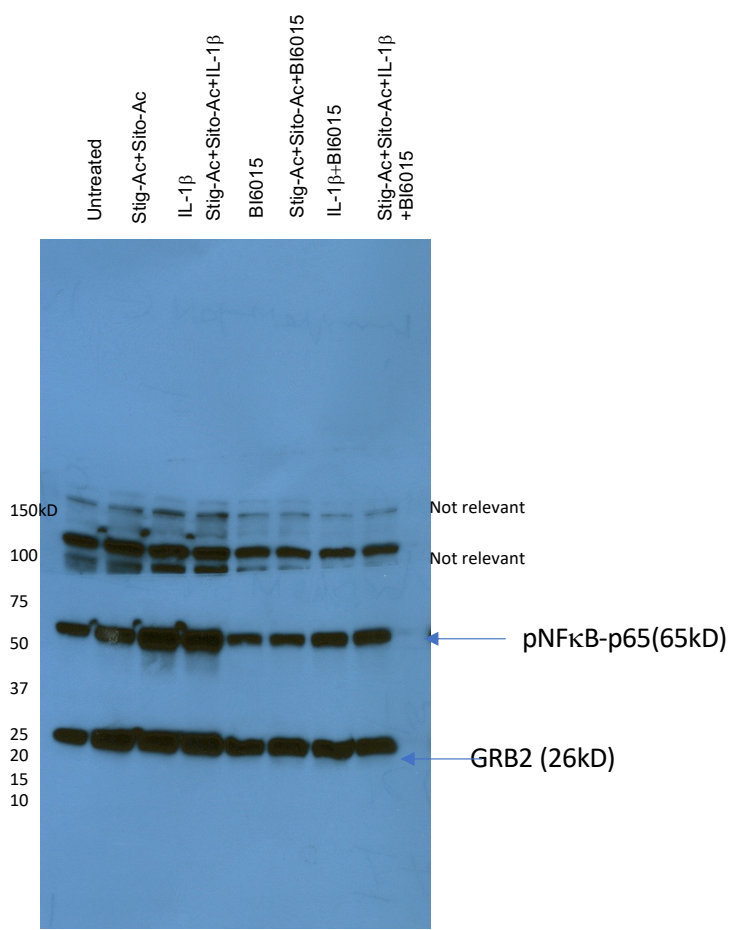

Figure 5F

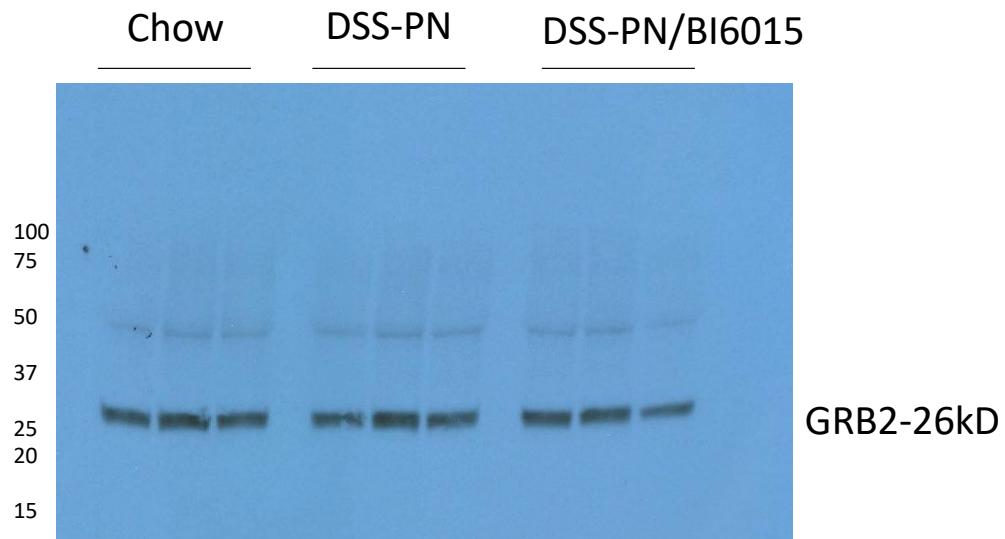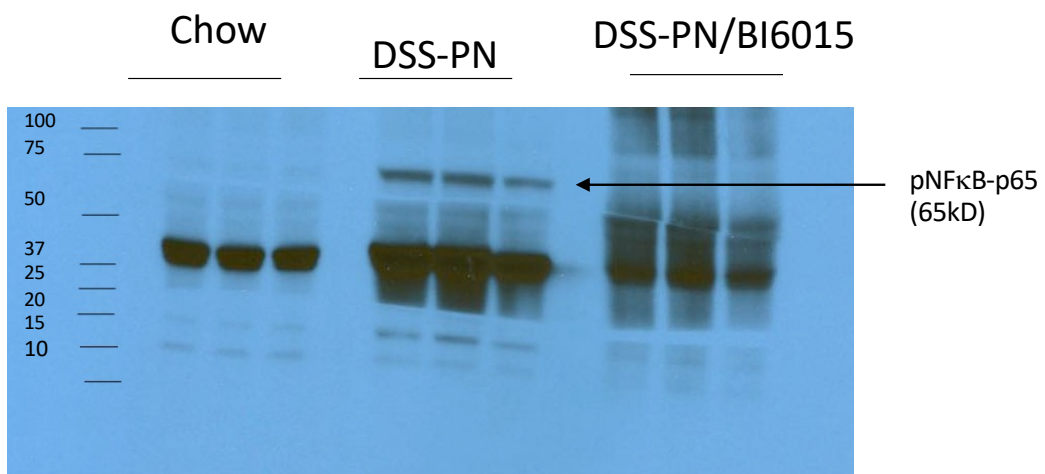

Figure 5F

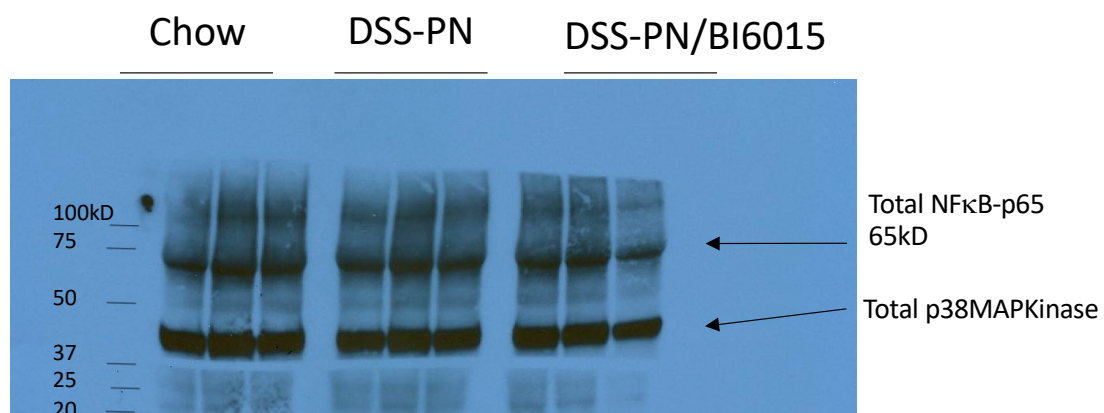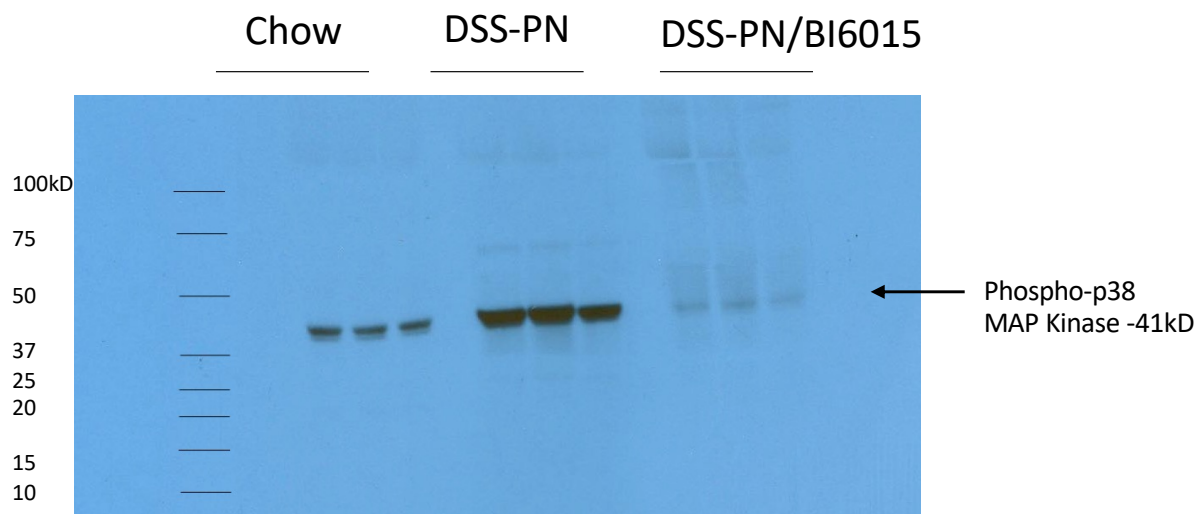

Supplement: Supplementary file 1 — Supplementary Information. [file 41598_2023_33994_MOESM1_ESM.pdf]
