# Supplementary material for: Pharmacologic inhibition of HNF4α prevents parenteral nutrition associated cholestasis in mice
Source: Sci Rep. 2023 May 12;13:7752. doi: 10.1038/s41598-023-33994-3 (PMC10182080; doi:10.1038/s41598-023-33994-3)
Supplement: Supplementary file 2 — Supplementary Table 1. [file 41598_2023_33994_MOESM2_ESM.docx]

**Supplementary Table 1. List of Antibodies**

| Antibody | Catalog No | Dilution | Vendor |
| --- | --- | --- | --- |
| HNF4α | ab41898 | 1:1000 (WB); 1:200(ICC) | Abcam |
| ABCG5 | Ab124965 | 1:1000 | Abcam |
| GRB2 | 3972 | 1:1000 | Cell Signaling |
| Total NFκB | 8242s | 1:1000 (WB)  1:200(ICC) | Cell Signaling |
| Phosphor-NFκB | 3033S | 1:1000 | Cell Signaling |
| Anti-Mouse- IgG-HRP | HAF007 | 1:5000 | R&D systems |
| Anti-Rabbit -IgG-HRP | 3972 | 1:5000 | Santa Cruz Biotechnology |
